# Clinical characteristics, predisposing factors and outcomes for *Enterococcus faecalis* versus *Enterococcus faecium* bloodstream infections: a prospective multicentre cohort study

**DOI:** 10.1007/s10096-024-04917-5

**Published:** 2024-08-08

**Authors:** Fenna Scharloo, Francesco Cogliati Dezza, Inmaculada López-Hernández, Pedro María Martínez Pérez-Crespo, Ane Josune Goikoetxea Aguirre, María Teresa Pérez-Rodríguez, Jonathan Fernandez-Suarez, Eva León Jiménez, Miguel Ángel Morán Rodríguez, Isabel Fernández-Natal, José María Reguera Iglesias, Clara Natera Kindelán, Maria Carmen Fariñas Álvares, Lucía Boix-Palop, Luis Eduardo Lopez-Cortes, Jesús Rodríguez-Baño, PROBAC/GEIRAS-SEIMC/SAMICEI group, Alfredo Jover-Sáenz, Juan Manuel Sánchez-Calvo, Isabel Gea-Lázaro, Alberto Bahamonde Carrasco, David Vinuesa García, Alfonso del Arco Jiménez, Alejandro Smithson Amat, Antonio Sánchez Porto, Inés Pérez Camacho, Jordi Cuquet Pedragosa, Esperanza Merino de Lucas, Berta Becerril Carral, Andrés Martín Aspas, Isabel Reche

**Affiliations:** 1 Faculty of Medicine, University Medical Center Utrecht, Utrecht, The Netherlands; 2 Department of Public Health and Infectious Diseases, Sapienza University of Rome, Rome, Italy; 3 Departamento de Medicina, Unidad Clínica de Enfermedades Infecciosas y Microbiología, Hospital Universitario Virgen Macarena, Universidad de Sevilla, Instituto de Biomedicina de Sevilla (IBiS)/CSIC, Seville, Spain; 4 CIBERINFEC, Instituto de Salud Carlos III, Madrid, Spain; 5 Unidad de Enfermedades Infecciosas y Microbiología, Hospital Universitario Nuestra Señora de Valme, Seville, Spain; 6 Unidad de Enfermedades Infecciosas, Hospital Universitario de Cruces, Bizkaia, Spain; 7 Departamento de Medicina Interna, Unidad de Enfermedades Infecciosas, Complexo Hospitalario Universitario de Vigo, Vigo, Spain; 8 Hospital Universitario Central de Asturias, Oviedo, Spain; 9 Unidad de Gestión Clínica de Enfermedades Infecciosas, Hospital Universitario de Burgos, Burgos, Spain; 10 Hospital Universitario de León, Complejo Asistencial Universitario de León, León, Spain; 11 Servicio de Enfermedades Infecciosas, Hospital Regional Universitario de Málaga, IBIMA Málaga, Málaga, Spain; 12 Unidad de Enfermedades Infecciosas, Hospital Universitario Reina Sofia, Cordoba, Spain; 13 Infectious Disease Service, Hospital Universitario Marqués de Valdecilla-IDIVAL, Universidad de Cantabria, Santander, Spain; 14 Infectious Diseases Department, Hospital Universitari Mútua Terrassa, Barcelona, Spain

**Keywords:** Bloodstream infection, Prognostic factors, Mortality, *Enterococcus faecalis*, *Enterococcus faecium*

## Abstract

**Purposes:**

Enterococcal BSI is associated with significant morbidity and mortality, with fatality rates of approximately 20–30%. There are microbiological and clinical differences between *E. faecalis* and *E. faecium* infections. The aim of this study was to investigate differences in predisposing factors for *E. faecalis* and *E. faecium* BSI and to explore prognostic factors.

**Methods:**

This study was a post-hoc analysis of PROBAC, a Spanish prospective, multicenter, cohort in 2016–2017. Patients with *E. faecalis* or *E. faecium* BSI were eligible. Independent predictors for BSI development in polymicrobial and monomicrobial BSI and in-hospital mortality in the monomicrobial group were identified by logistic regression.

**Results:**

A total of 431 patients were included. Independent factors associated with *E. faecium* BSI were previous use of penicillins (aOR 1.99 (95% CI 1.20–3.32)) or carbapenems (2.35 (1.12–4.93)), hospital-acquired BSI (2.58 (1.61–4.12)), and biliary tract source (3.36 (1.84–6.13)), while congestive heart failure (0.51 (0.27–0.97)), cerebrovascular disease (0.45 (0.21–0.98)), and urinary tract source (0.49 (0.26–0.92)) were associated with *E. faecalis* BSI. Independent prognostic factors for in-hospital mortality in *E. faecalis* BSI were Charlson Comorbidity Index (1.27 (1.08–1.51)), SOFA score (1.47 (1.24–1.73)), age (1.06 (1.02–1.10)), and urinary/biliary source (0.29 (0.09–0.90)). For *E. faecium* BSI, only SOFA score (1.34 (1.14–1.58) was associated with in-hospital mortality.

**Conclusions:**

The factors associated with *E. faecium* and *E. faecalis* BSI are different. These variables may be helpful in the suspicion of one or other species for empiric therapeutic decisions and provide valuable information on prognosis.

**Supplementary Information:**

The online version contains supplementary material available at 10.1007/s10096-024-04917-5.

## Introduction

Enterococci are gram-positive cocci that are part of the commensal flora of the gut [1]. The two most common species, *Enterococcus faecium* and *Enterococcus faecalis*, are important causes of bloodstream infection (BSI) [1], *Enterococcus* is currently the second most common genus of causative pathogens of gram-positive BSI in Europe and the United States, and the incidence may be increasing [1–3]. Enterococcal BSI is associated with significant morbidity and mortality. Several studies have shown mortality rates of approximately 20–30% [4–7]. The population at risk comprises mostly fragile patients, in particular, patients with multiple co-morbidities, prolonged hospitalisation stay, elderly patients, and immunocompromised patients [1, 5, 8].

*E. faecalis* and *E. faecium* infections show great differences in terms of prevalence, resistance, and patient characteristics, despite belonging to the same genus. In terms of BSI prevalence, *E. faecalis* is the predominant pathogen compared to *E. faecium*, although the distribution varies between countries [5, 6, 9, 10]. As for resistance profile, *E. faecalis* are typically susceptible to ampicillin with only few exceptions, while *E. faecium* is frequently resistant to ampicillin [6]. Vancomycin resistance is more frequently found in *E. faecium*, which can be a major challenge in clinical management [1, 11]. Regarding the source of infection, *E. faecium* has been more frequently associated with a gastrointestinal source, while *E. faecalis* is mostly associated with a genitourinary focus [5], and is more frequently a cause of endocarditis [11]. Several published studies have also observed more severe comorbidities and higher mortality rates in *E. faecium* BSI compared to *E. faecalis* BSIs [5, 6, 9].

Since enterococcal BSI is associated with significant mortality, and the population at risk may be increasing, it is crucial to gain a deeper understanding of this disease. Furthermore due to the different resistance profiles, the choice of empirical therapy is a difficult challenge, leading to a low rate of appropriate empirical therapy, a known protective factor in the BSI prognosis [11]. It is crucial therefore to be aware of the differences between the species in order to properly assess the probability of *E. faecium* or *E. faecalis* as the BSI cause and to help decide on an early appropriate empirical therapy.

The aims of this study therefore were (I) to investigate the differential predisposing factors associated with either *E. faecalis* or *E. faecium* as the cause of BSI in patients with BSI due to *Enterococcus* spp., and (II) to explore the prognostic predictors of in-hospital mortality, and specifically whether either of the two main *Enterococcus* species was associated with in-hospitality, to add to the knowledge on enterococcal BSI.

## Materials and methods

### Study design and population

This study is a post hoc analysis of the PROBAC project. PROBAC is a national multicentre, observational, prospective cohort study conducted in 26 Spanish hospitals (18 tertiary and 8 community hospitals) between October 2016 and March 2017 [3]. The PROBAC cohort included all episodes of clinically significant BSI in patients aged ≥ 14 years; the only exclusion criterion was isolation of a typical contaminant (e.g., coagulase-negative staphylococci, diphtheroids) in blood cultures. Patients were followed during hospitalisation for a maximum of 30 days after the onset of BSI. Additional information about the PROBAC study design has been published previously [3, 12].

For this sub-analysis, patients in the PROBAC cohort with BSI caused by *E. faecalis* or *E. faecium* were eligible, hereafter referred to as enterococcal BSI. To assess predisposing factors for development of *E. faecalis* or *E. faecium* BSI, both polymicrobial and monomicrobial enterococcal BSIs were included. To analyse prognostic factors associated with *E. faecalis* BSI and *E. faecium* BSI, only monomicrobial enterococcal BSIs were included. The monomicrobial study population was divided into two groups (*E. faecalis* BSI and *E. faecium* BSI) to better explore differences in clinical characteristics and outcomes of these subpopulations.

### Data collection

Data were collected using an anonymous electronic case report form. Variables collected included demographics and clinical data concerning comorbidities; immunosuppressive therapy; previous antibiotic use; invasive procedures and exposure to devices in the previous month; clinical severity at onset of BSI; type of acquisition and source of infection. Microbiological variables included *Enterococcus* species (*E. faecium* or *E. faecalis*) and resistance profile.

### Endpoints

The endpoint for identifying predisposing factors for the two *Enterococcus* species was isolation of *E. faecalis* or *E. faecium* in blood cultures. The primary endpoint for the outcome analysis was all cause in-hospital mortality (up to day 30); secondary endpoints were 30-day, in-hospital, BSI-related mortality, fever lasting ≥ 72 h, persistent bacteremia and recurrent infection.

### Definitions

Bloodstream infection was defined as one or more positive blood cultures with signs or symptoms of infection. Subsequent BSI episodes due to same pathogen in the same patient were excluded if they occurred < 3 months apart. Blood cultures were collected, processed, and interpreted according to standard microbiological practices at each centre.

Type of acquisition was defined as hospital-acquired (if the BSI occurred > 48 h after hospital admission), healthcare-associated (according to predefined criteria [13]), or community-acquired (if neither hospital-acquired and healthcare-associated were applicable).

Charlson Comorbidity Index (CCI) [14] was used to classify the burden of comorbidity. The CCI used in the analysis was not adjusted for age, as age was included as an independent variable. Clinical severity at BSI onset was determined through data from Sequential Organ Failure Assessment (SOFA) score [15], Pitt score [16], and septic shock. Septic shock was defined according to the third international consensus definition [17]. Source of infection was assessed according to US Centers for Disease Control and Prevention definition [18]. Immunosuppressive therapy included antineoplastic chemotherapy in the previous month, and prednisone (or equivalent) at doses > 10 mg/day for more than 3 weeks.

Empirical therapy was defined as therapy administered prior to the availability of microorganism susceptibility tests. It was considered active if the isolated pathogen showed susceptibility to the empiric therapy used.

All cause in-hospital mortality was defined as death due to any cause during hospitalisation within 30 days of BSI onset. Enterococcal BSI- related mortality was defined as death during this period that, in the opinion of the investigator, was directly caused by the infection or its complications, in the absence of other probable causes.

### Statistical analysis

Categorical variables were presented as frequency counts with percentages. Continuous variables were presented as median with interquartile ranges (IQR). Continuous variables were compared using Student’s t-test or Mann–Whitney U test, as appropriate. Categorical variables were compared using Chi-squared or Fisher’s exact test, as appropriate. Mortality risks were presented as relative risks with 95% confidence intervals (CI).

Missing data were reported; patients with missing values in critical data were excluded from the multivariable analysis. No additional analysis were performed to handle missing data.

Multivariable logistic regression model was performed to explore the independent predictors of BSI aetiology (*E. faecalis* or *E. faecium*) and 30-day mortality in patients with *E. faecalis* BSI and *E. faecium* BSI. Disease progression factors were not included as variables in the multivariable analysis to avoid possible survival bias in these models. Therefore, clinically meaningful variables for the selected outcomes with a univariate P value < 0.2 were included in the multivariable analysis. For the mortality analysis, possible confounders and effect modifiers were identified and also included in the multivariable analysis. Multicolinearty was assessed and reported in Supplementary Material (Table S1). In the first step, the included variables were based on the causal assumptions as considered in a directed acyclic graph (DAG) model which was included in a recently published systematic review and Delphi consensus on universal risk factors for mortality in BSI [19] Next, the selection of variables was performed by a stepwise backward procedure guided by DAG. Notably, we included recent invasive procedures because they may capture the underlying conditions of patients that would otherwise be neglected. P-values were two-tailed and a P value of < 0.05 was considered statistically significant. Multivariable models were assessed for suitability using the Hosmer–Lemeshow goodness of fit test and area under the receiver operating characteristic curve. Data were analysed using IBM SPSS Statistics v29.0 (IBM Corp., Armonk, NY, USA).

### Ethical approval

The PROBAC study was approved by the ethics committees of the participating centres. The need for informed consent was waived due to the observational design. PROBAC is registered in Clinicaltrials.gov (NCT03148769). For this post hoc analysis, no additional approval was necessary.

## Results

### Participants

**Fig. 1:**
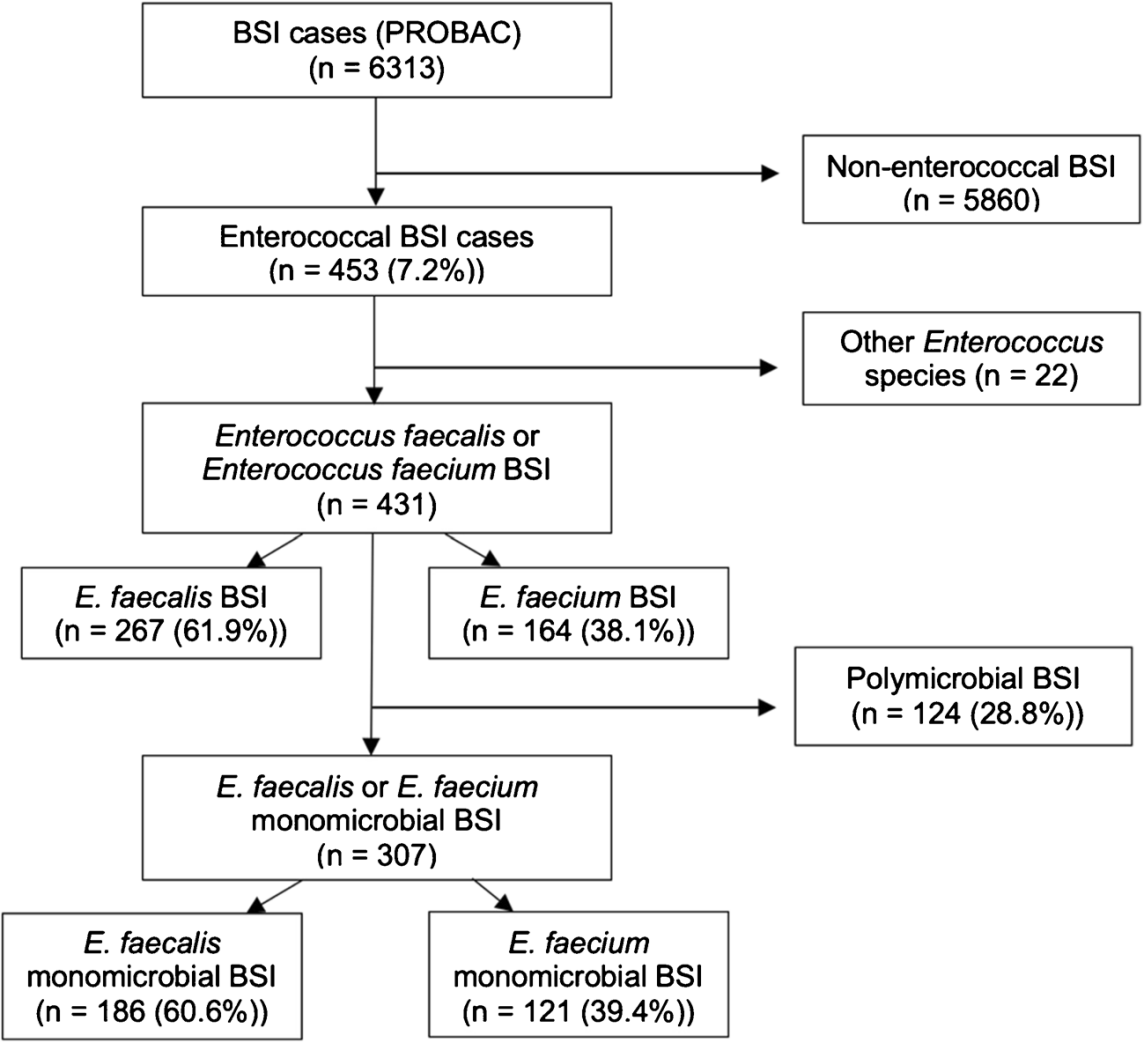
Flow chart of study population. For the first objective (differences between species), both poly- and monomicrobial BSI were used (*n* = 431). For the second objective (outcome analysis), only monomicrobial BSI were used (*n* = 307)

The PROBAC cohort included 6313 BSI episodes, of which 431 patients (6.8%) had a BSI caused by *E. faecalis* or *E. faecium.* All 431 patients were included in the study of differential characteristics between the two species. *E. faecalis* was the predominant causative pathogen (61.9%). Polymicrobial infections accounted for 28.8% of all enterococcal BSIs, and the 307 patients with monomicrobial enterococcal BSI (186 due to *E. faecalis* and 121 due to *E. faecium*) were included in the outcome analysis. The study flow chart is shown in Fig. 1.

### Characteristics of enterococcal BSI

The main characteristics of patients with enterococcal BSI and the differences between *E. faecalis* and *E. faecium* BSI are shown in Tables 1. Median age was 72 (IQR 62 -82), 66.9% were male. The median CCI was 3 (1–5) for both the *E. faecalis* and *E. faecium* BSI populations. Congestive heart failure, cerebrovascular disease and obstructive uropathy were more frequent among *E. faecalis* BSI*.* By contrast, hepatic disease, solid tumour and obstructive biliary pathology were more frequently associated with *E. faecium* BSI*.*

**Table 1:** General characteristics and outcomes of all *E. faecalis* and *E. faecium* bloodstream infection

	**All BSI (*n* = 431)**	**E. faecalis BSI (*n* = 267)**	**E. faecium BSI (*n* = 164)**	***p*-value**
**Demographic**				
Age, median [IQR]	72 [62—82]	72 [63—83]	69 [60—81]	0.097^γ^
Male sex, n (%)	287 (66.9)	183 (68.8)	104 (63.8)	0.286
**Comorbidities, n (%)**				
CCI, median (IQR)	3 [1-5]	3 [1-5]	3 [1-5]	0.786
Congestive heart failure^a^	78 (18.1)	61 (22.8)	17 (10.4)	** < 0.001**^**γ**^
Diabetes Mellitus	114 (26.5)	73 (27.3)	41 (25.0)	0.593
Chronic kidney disease^b^	72 (16.7)	45 (16.9)	27 (16.5)	0.916
Hepatic disease^c^	51 (11.8)	24 (9.0)	27 (16.5)	**0.020**^**γ**^
Solid tumor	133 (30.9)	70 (26.2)	63 (38.4)	**0.008**^**γ**^
Cerebrovascular disease^d^	51 (11.8)	40 (15.0)	11 (6.7)	**0.010**^**γ**^
Hematologic malignancy	34 (7.9)	24 (9.0)	10 (6.1)	0.280
Obstructive uropathy	28 (6.5)	23 (8.6)	5 (3.0)	**0.023**^**γ**^
Obstructive biliary pathology	29 (6.7)	10 (3.7)	19 (11.6)	**0.002**^**γ**^
Immunosuppressive therapy^e^	51 (11.8)	30 (11.2)	21 (12.8)	0.624
**Invasive procedures (in the previous month), n (%)**				
Surgery	77 (17.9)	40 (15.0)	37 (22.6)	**0.046**^**γ**^
Bronchoscopy	41 (9.5)	18 (6.7)	23 (14.0)	**0.012**^**γ**^
Urinary catheter	100 (23.2)	61 (22.8)	39 (23.8)	0.824
**Use of antibiotics (in the previous month), n (%)**				
Any antibiotic	189 (43.9)	100 (37.5)	89 (54.3)	** < 0.001**
Cephalosporins	64 (14.8)	38 (14.2)	26 (15.9)	0.646
Penicillins	100 (23.3)	45 (16.9)	55 (33.5)	** < 0.001**^**γ**^
Carbapenems	42 (9.7)	14 (5.2)	28 (17.1)	** < 0.001**^**γ**^
Vancomycin, linezolid or daptomycin	47 (10.9)	21 (7.9)	26 (15.9)	**0.010**^**γ**^
Quinolones	56 (13.0)	36 (13.5)	20 (12.2)	0.699
**Type of acquisition, n (%)**				
Hospital-acquired	221 (52.5)	110 (42.3)	111 (68.9)	** < 0.001**^**γ**^
Healthcare-associated	103 (24.5)	70 (26.9)	33 (20.5)	0.136^**γ**^
Community-acquired	97 (23.0)	80 (30.8)	17 (10.6)	** < 0.001**^**γ**^
Onset in intensive care unit	54 (12.9)	25 (9.7)	29 (18.0)	**0.013**^**γ**^
**Source of infection, n (%)**				
Biliary tract	72 (16.7)	25 (9.4)	47 (28.7)	** < 0.001**^**γ**^
Abdominal (non-biliary)	52 (12.1)	25 (9.4)	27 (16.5)	**0.028**^**γ**^
Catheter-related	41 (9.5)	26 (9.7)	15 (9.1)	0.839
Endocarditis	26 (6.0)	24 (9.0)	2 (1.2)	**0.001**^**γ**^
Bone and joint	1 (0.2)	1 (0.4)	0 (0.0)	1.000
Skin and soft tissue	8 (1.9)	4 (1.5)	4 (2.4)	0.486
Respiratory	16 (3.7)	14 (5.2)	2 (1.2)	**0.032**^**γ**^
Central nervous system	4 (0.9)	3 (1.1)	1 (0.6)	1.000
Urinary tract	84 (19.5)	68 (25.5)	16 (9.8)	** < 0.001**^**γ**^
Other	4 (0.9)	1 (0.4)	3 (1.8)	0.156^**γ**^
Unknown	123 (28.5)	76 (28.5)	47 (28.7)	0.965
**Microbiology, n (%)**				
Polymicrobial	124 (28.8)	81 (30.3)	43 (26.2)	0.359
Ampicillin resistance^#^	162 (45.9)	40 (18.0)	122 (93.1)	** < 0.001**
Vancomycin resistance^#^	10 (3.2)	1 (0.5)	9 (7.1)	0.002*
**Clinical presentation, n (%)**				
Septic shock	47 (10.9)	24 (9.0)	23 (14.0)	0.103
Pitt score, median [IQR]	1 [ 0–3]	1 [0 – 3]	1 [0 – 3]	0.574
SOFA score^#^, median [IQR]	2 [0 – 4]	2 [0 – 4]	2 [0—4]	0.659
**Outcome, n (%)**				
In-hospital mortality	100 (23.2)	64 (24.0)	36 (22.0)	0.630
BSI-related mortality	60 (13.9)	36 (13.5)	24 (14.6)	0.738

*Abbreviations*: *BSI* bloodstream infection, *CCI* Charlson comorbidity index, *NA* Not applicable, *SOFA* Sequential Organ Failure Assessment

^a^Congestive heartfailure: stage III or IV according to the New York Heart Association

^b^Chronic kidney disease: moderate to severe kidney disease, for more than 1 month

^c^Hepatic disease: includes mild (chronic hepatitis) and severe (cirrhosis and portal hypertension with history of variceal bleeding) hepatic disease

^d^Cerebrovascular disease: history of cerebrovascular accident with minor/no residual deficits or transient ischemic attacks

^e^Immunosuppressive therapy included antineoplastic chemotherapy and prednisone (or equivalent) at doses > 10 mg/day for more than 3 weeks

^γ^ Variables used with stepwise-backward multivariabe analysis. In addition to these variables, recurrent urinary tract infection (p 0.078) and colonoscopy in previous month (p 0.193) were also used in the initial model of the multivariable analysis

^#^Missing data: the following variables were available for a proportion of the patients: ampicillin resistance (n = 353/431); vancomycin resistance (*n* = 311/431)

The majority of BSIs were hospital-acquired (42.3% vs 68.9% *p* < 0.001). Regarding the source of infection, a urinary tract source and endocarditis were more common in *E. faecalis* BSI compared to *E. faecium* BSI. *E. faecium* BSI was more frequently associated with a biliary tract source or an abdominal source. Unknown source was highly prevalent in both groups (28.5%).

There were no differences in clinical severity; at diagnosis, the median SOFA score was 2 (0—4), and the median Pitt score was 1 (0—3) for both groups.

### Predisposing factors for BSI due to *E. faecalis* or *E. faecium* in patients with enterococcal BSI

The variables associated with *E. faecium* BSI rather than *E. faecalis* by multivariable analysis were use in the previous 30 days of penicillins (aOR 1.99 (95% CI 1.20–3.32), *p* = 0.008) or carbapenems (aOR 2.35 (1.12–4.93), *p* = 0.025), hospital-acquired BSI (aOR 2.58 (1.61–4.12), p < 0.001), and biliary tract source (aOR 3.36 (1.84–6.13) *p* < 0.001). By contrast, congestive heart failure (aOR 0.51 (0.27–0.97), *p* = 0.039), cerebrovascular disease (aOR 0.45 (0.21–0.98), *p* = 0.045), and urinary tract source (aOR 0.49 (0.26–0.92), *p* = 0.028) were independent “protective” factors for *E. faecium* BSI, or associated with *E. faecalis* BSI (Table 2).

**Table 2:** Multivariable analysis for predictors of bloodstream infection due to *E. faecium* instead of *E. faecalis*

	**B -coefficient**	**aOR (95% CI)**	***p*-value**
Congestive heart failure^a^	-0.671	0.51 (0.27 – 0.97)	**0.039**
Cerebrovascular disease^b^	-0.783	0.45 (0.21 – 0.98)	**0.045**
Use of penicillins in previous month	0.689	1.99 (1.20 – 3.32)	**0.008**
Use of carbapenems in previous month	0.852	2.35 (1.12 – 4.93)	**0.025**
Hospital-acquired infection	0.947	2.58 (1.61 – 4.12)	** < 0.001**
Biliary source	1.212	3.36 (1.84 – 6.13)	** < 0.001**
Urinary source	-0.714	0.49 (0.26 – 0.92)	**0.028**

*aOR* adjusted OR, displayed as predisposing factors to *E. faecium*, in reference to *E. faecalis*

^a^Congestive heart failure: stage III or IV according to the New York Heart Association

^b^Cerebrovascular disease: history of a cerebrovascular accident with minor/no residue or transient ischemic attacks

Area Under the Receiver Operating Curve: 0.74 (95% CI 0.70—0.79)

Due to the small number of cases, endocarditis was not included in the model. However, we constructed an alternative model (not shown) in which endocarditis source was added to the previous predictors. This resulted in a similar model in which endocarditis showed a non-significant association with *E. faecalis* BSI (aOR for *E. faecium*, 0.28 (0.06–1.27),* p* = 0.098).

### Monomicrobial enterococcal BSI

Three hundred and seven enterococcal BSIs were monomicrobial (71.2%), including 186 *E. faecalis* BSIs (60.6%) and 121 *E. faecium* BSIs (39.4%). Table S2 shows the characteristics of the monomicrobial enterococcal BSI population. Regarding outcome, 64 patients (20.8%) died during hospitalisation; in-hospital mortality was similar for *E. faecalis* and *E. faecium* (crude comparison, 20.4% vs 21.5%, respectively, *p* = 0.824). There were no differences in BSI-related mortality between the two groups (11.3% (21) vs 11.6% (14), *p* = 0.940). Persistent BSI occurred in 5.9% (11 patients) and 5.0% (6*)* (*p* = 0.721). Recurrence of infection was more frequent in *E. faecium* BSI, although the difference was not statistically significant. Other outcomes are shown in Table S2.

### Predictors of in-hospital mortality in enterococcal BSI

Table S3 in Supplementary Material shows the bivariate analysis of factors associated with mortality in the monomicrobial enterococcal BSI population. Age (aOR 1.03 (95% CI 1.00–1.06), *p* = 0.031), CCI (aOR 1.16 (1.03–1.31), *p* = 0.013), use of urinary catheter (aOR 2.69 (1.31–5.53), *p* = 0.007) and SOFA score (aOR 1.34 (1.17–1.53), *p* < 0.001) were selected as predictors of in-hospital mortality in multivariate analysis. However, the *Enterococcus* species (*E. faecium* vs *E. faecalis*) was not found to be associated with in-hospital mortality (aOR 1.06 (0.55 – 2.07), *p* = 0.861), nor was Pitt score (Table 4A).

In a multivariable model of BSI-related mortality (not shown), *Enterococcus* species (*E. faecium* vs *E. faecalis*) was not found to be significantly associated after controlling for age, sex, congestive heart failure, diabetes mellitus and SOFA score (aOR 1.41 (0.59–3.40), *p* = 0.443).

### Mortality in *E. faecalis *BSI and *E. faecium* BSI

The bivariate analysis of factors associated with in-hospital mortality in the *E. faecalis* BSI population is shown in Table 3A. In the multivariable model (Table 4B), CCI (aOR 1.27 (95% CI 1.08–1.51), p = 0.005), SOFA score (1.47 (1.24–1.73), p < 0.001) and age (1.06 (1.02–1.10) p = 0.004) were selected as independent predictors, while urinary or biliary source was found to be a protective factor (aOR 0.29 (0.09–0.90) p = 0.031).

**Table 3:** Bivariate analysis of 30-day all cause in-hospital mortality^a^ for *E. faecalis* bloodstream infection (A, left side) and *E. faecium* bloodstream infection (B, right side)

	**A. *E. faecalis* BSI (*n* = 186)**				**B. *E. faecium* BSI (*n* = 121)**			
	**Mortality** ^a^ **with factor**	**Mortality without factor**	**RR (95% CI)**	***p*-value**	**Mortality with factor**	**Mortality without factor**	**RR (95% CI)**	***p*-value**
**Demographic**								
Age ≥ 69 years	28/110 (25.5)	10/76 (13.2)	1.93 (1.00 – 3.75)	**0.041**^**γ**^	14/60 (23.3)	12/60 (20.0)	1.17 (0.59—2.31)	0.658
Male sex	27/123 (22.0)	10/62 (16.1)	1.36 (0.70—2.63)	0.350	17/76 (22.4)	9/44 (20.5)	1.09 (0.53 – 2.24)	0.806
**Comorbidities, n (%)**								
CCI ≥ 3	30/93 (32.3)	8/93 (8.6)	3.75 (1.81—7.75)	** < 0.001**^γ^	14/58 (24.1)	12/63 (19.0)	1.27 (0.65 – 2.51)	0.496
Congestive heart failure^b^	17/48 (35.4)	21/138 (15.2)	2.33 (1.34 – 4.03)	**0.003**^**γ**^	3/15 (20.0)	23/106 (21.7)	0.92 (0.32- 2.70)	1.000*
Diabetes mellitus	13/47 (27.7)	25/139 (18.0)	1.54 (0.86 – 2.75)	0.155^**γ**^	7/27 (25.9)	19/94 (20.2)	1.28 (0.60—2.72)	0.524
Peptic ulcer disease	4/7 (57.1)	34/179 (19.0)	3.01 (1.48 – 6.10)	0.033*	3/7 (42.9)	23/114 (20.2)	2.12 (0.84 – 5.38)	0.169*^γ^
Urinary catheter	10/36 (27.8)	28/150 (18.7)	1.49 (0.80 – 2.78)	0.223	11/30 (36.7)	15/91 (16.5)	2.22 (1.15 – 4.31)	**0.020**^**γ**^
Antibiotic use (past month)	14/63 (22.2)	24/123 (19.5)	1.14 (0.63- 2.04)	0.664	19/63 (30.2)	7/58 (12.1)	2.50 (1.13 – 5.49)	**0.016**^**γ**^
**Type of acquisition, n (%)**								
Hospital-acquired	15/66 (22.7)	22/113 (19.5)	1.17 (0.65 – 2.09)	0.604	22/89 (24.7)	4/29 (13.8)	1.79 (0.67 – 4.76)	0.218
Healthcare-associated	9/56 (16.1)	28/123 (22.8)	0.71 (1.39 – 0.36)	0.305	4/22 (18.2)	22/96 (22.9)	0.79 (0.30 – 2.07)	0.779*
Community-acquired	13/57 (22.8)	24/122 (19.7)	1.16 (0.64 – 2.11)	0.629	0/7 (0.0)	26/111 (23.4)	-	0.345*
Onset in Intensive care unit^#^	5/18 (27.8)	33/163 (20.2)	1.37 (0.61 – 3.07)	0.541*	11/26 (42.3)	15/93 (16.1)	2.62 (1.38 – 5.00)	**0.004**^**γ**^
**Source of infection, n (%)**								
Biliary tract	0/8 (0.0)	38/178 (21.3)	NA	0.363*	6/28 (21.4)	20/93 (21.5)	1.00 (0.44 – 2.24)	0.993
Abdominal (non-biliary)	4/18 (22.2)	34/168 (20.2)	1.10 (0.44 – 2.74)	0.766*	6/21 (28.6)	20/100 (20.0)	1.43 (0.65 – 3.13)	0.390*
Catheter-related	4/13 (30.8)	34/173 (19.7)	1.56 (0.66 – 3.73)	0.307*	2/8 (25.0)	24/113 (21.2)	1.18 (0.34 – 4.12)	0.681*
Endocarditis	5/23 (21.7)	33/163 (20.2)	1.07 (0.47 – 2.47)	0.789*	0/2 (0.0)	26/119 (21.8)	NA	1.000*
Bone and joint	0/0 (0.0)	38/186 (20.4)	NA	NA	0/0 (0.0)	26/121 (21.5)	NA	NA
Skin and soft tissue	1/2 (50.0)	37/184 (20.1)	2.49 (0.60 – 10.2)	0.368*	1/3 (33.3)	25/118 (21.2)	1.57 (0.31 – 8.06)	0.519*
Respiratory	3/9 (33.3)	35/177 (19.8)	1.69 (0.64 – 4.44)	0.392*	0/1 (0.0)	26/120 (21.7)	NA	1.000*
Urinary tract	8/49 (16.3)	30/137 (21.9)	0.75 (0.37 – 1.52)	0.406	3/11 (27.3)	23/110 (20.9)	1.30 (0.47 – 3.66)	0.701*
Central nervous system	0/1 (0.0)	38/185 (20.5)	NA	1.000*	0/0 (0.0)	26/121 (21.5)	NA	NA
Other	0/1 (0.0)	38/185 (20.5)	NA	1.000*	0/2 (0.0)	26/119 (21.8)	NA	1.000*
Unknown	13/62 (21.0)	25/124 (20.2)	1.04 (0.57 – 1.89)	0.898	8/45 (17.8)	18/76 (23.7)	0.75 (0.36 – 1.58)	0.445
**Microbiology, n (%)**								
Ampicillin resistance^#^	2/6 (33.3)	30/157 (19.1)	7.62 (0.53 – 5.65)	0.335*	20/95 (21.1)	1/6 (16.7)	1.26 (0.20 – 7.87)	1.000*
Vancomycin resistance^#^	0/0 (0.0)	31/154 (20.1)	NA	NA	1/8 (12.5)	22/101 (21.8)	0.57 (0.09 – 3.72)	1.000*
Active empirical antibiotic	20/102 (19.6)	18/84 (21.4)	0.91 (0.52 – 1.61)	0.759	14/42 (33.3)	12/79 (15.2)	2.19 (1.12 – 4.31)	**0.021**^**γ**^
Active targeted antibiotic^#^	23/140 (16.4)	4/12 (33.3)	0.49 (0.20 – 1.19)	0.228*	17/99 (17.2)	2/4 (50.0)	0.34 (0.12 – 1.00)	0.154*
**Clinical presentation, n (%)**								
Septic shock	7/12 (58.3)	31/174 (17.8)	3.28 (1.84 – 5.81)	** < 0.001**	7/15 (46.7)	19/106 (17.9)	2.60 (1.32 – 5.13)	0.019*
Pitt score ≥ 3	19/48 (39.6)	19/138 (13.8)	2.87 (1.67 – 4.95)	**0.002**^**γ**^	10/27 (37.0)	16/94 (17.0)	2.17 (1.12 – 4.22)	**0.026**^**γ**^
SOFA ≥ 3	27/71 (38.0)	8/111 (7.2)	5.26 (2.54 –11.0)	** < 0.001**^**γ**^	16/54 (29.6)	8/65 (12.3)	2.41 (1.12 – 5.18)	**0.019**^**γ**^

*Abbreviations*: *BSI* blood stream infection, *CCI* Charlson comorbidity index, *NA* Not applicable, *SOFA* Sequential Organ Failure Assessment

^*^calculated by Fisher’s exact test, all other p-values are calculated by chi-squared

^a^Mortality: during hospitalisation, with a maximum of 30 days after BSI onset

^b^Congestive heart failure: stage III or IV according to the NYHA

^γ^V ariables used with stepwise-backward multivariabe analysis. In addition to these variables, for *E. faecalis* BSI, chronic kidney disease (p 0.001), hepatic disease (p 0.101), cerebrovascular disease (p 0.159), hemiplegy (p 0.072), presence of a pacemaker/ICD (p 0.101), presence of peripheric venous catheter (p 0.193) were used in the initial model for multivariable analysis. For *E. faecium* BSI, hepatic disease (p 0.108), use of immunosuppressants (p 0.108), use of parenteral feeding (p 0.178), medical ward (p 0.026) were additionally used in the initial model for multivariable analysis

^#^Missing data: the following variables were available for a part of the patients;ampicillin resistance (n = 163/186 (A) and n = 101/121 (B)); vancomycin resistance (n = 154/186 (A) and 109/121 (B)); active targeted antibiotics (n = 152/186 (A) and 103/121 (B)

**Table 4:** Multivariable models of factors associated with in-hospital mortality. Monomicrobial enterococcal bloodstream infection (A); Monomicrobial *E. faecalis* bloodstream infection (B); Monomicrobial E. faecium bloodstream infection (C)

**A**	**B-coefficient**	**aOR (95% CI)**	***p*-value**
*E. faecium* (reference: *E. faecalis*)	0.087	1.06 (0.55—2.07)	0.861
Age^a^	0.849	1.03 (1.00 – 1.06)	**0.031**
Charlson Comorbidity Index^b^	0.947	1.16 (1.03 – 1.31)	**0.013**
Urinary catheter	0.845	2.69 (1.31 – 5.53)	**0.007**
SOFA score^a^	1.414	1.34 (1.17 – 1.53)	** < 0.001**
Pitt score	0.624	1.87 (0.93 – 1.26)	0.304
**B**	**B-coefficient**	**aOR (95% CI)**	***p*-value**
Age^a^	0.058	1.06 (1.02–1.10)	**0.004**
Charlson Comorbidity Index^b^	0.242	1.27 (1.08–1.51)	**0.005**
SOFA score^b^	0.383	1.47 (1.24–1.73)	** < 0.001**
Urinary or biliary source	-1.239	0.29 (0.09–0.90)	**0.031**
**C**	**B-coefficient**	**aOR (95% CI)**	***p-*value**
Age^a^	-0.007	0.99 (0.95—1.03)	0.731
Charlson Comorbidity Index^b^	-0.052	0.95 (0.79 – 1.14)	0.586
SOFA score^b^	0.294	1.34 (1.14 – 1.58)	** < 0.001**
Urinary catheter	1.082	2.95 (0.96 – 9.11)	0.060
Antibiotic use in previous month	1.159	3.19 (0.96 – 10.60)	0.059

*Abbreviations*: *BSI* bloodstream infection, *aOR* adjusted OR; SOFA, Sequential Organ Failure Assessment

Area Under the Receiver Operating Curve: A: 0.81 (95% CI 0.75—0.87); B: 0.85 (0.78—0.93); C: 0.80 (0.70—0.90)

^a^Per year

^b^Per index unit

Table 3B shows the bivariate analysis of factors associated with in-hospital mortality in the *E. faecium* BSI population. In the multivariable model (Table 4C), SOFA score (aOR 1.34; 95% CI 1.14–1.58; *p* < 0.001), use of antibiotics in the previous month (aOR 3.19; 95% CI 0.96–10.60; *p* = 0.059) and urinary catheter (aOR 2.95; 0.96–9.11, p = 0.060) were associated with mortality, although the last two were not statistically significant. Of note, in *E. faecium* BSI, CCI and age were not significantly associated with in-hospital mortality.

## Discussion

In this study, we found that the predisposing factors associated with *E. faecium* compared to *E. faecalis* in patients with BSI due to *Enterococcus* spp were different. Previous use of penicillins or carbapenems, hospital-acquired BSI and biliary-tract source were independently associated with *E. faecium* BSI, whereas congestive heart failure, cerebrovascular disease and urinary tract source were independent factors associated with *E. faecalis* BSI. We found an in-hospital mortality rate of approximately 21% in monomicrobial enterococcal BSI, with no differences between *E. faecalis* and *E. faecium*. We further observed that while in-hospital mortality in episodes caused by *E. faecium* was mostly predicted by clinical severity at the onset of BSI, several other factors, including the burden of comorbidities and age were associated with higher risk of death in episodes caused by *E. faecalis*. In addition, urinary or biliary source was identified as a protective factor only for *E. faecalis*. To the best of our knowledge, this is the only prospective multicentre cohort study on both predisposing and prognostic factors in *E. faecium* and *E. faecalis* BSI.

Overall, the enterococcal BSI population in this study was mainly elderly people with a high burden of comorbidities, as shown in previous studies [5, 20]. Regarding the enterococcal species causing BSI, the proportion of *E. faecium* was higher than in studies from the previous decade, also performed in Spain, which reported *E. faecium* rates of 26% and 12% [4, 20]. This increase reflects a trend, previously described, which has also been linked to the higher intrinsic antimicrobial resistance of *E. faecium* [1, 21].

Our study confirmed differences in the predominant sources of infection between *E. faecalis* and *E. faecium* that were already known [5, 6, 9, 11]. Urinary source and endocarditis were more prevalent in *E. faecalis* BSI, while abdominal and biliary source were predominant in *E. faecium*. There was a high prevalence of patients with unknown source of BSI, especially in monomicrobial enterococcal BSI, in agreement with previous retrospective studies [6, 22].

The multivariable models for the different predisposing factors for enterococcal species supported some of the predictors found in previous retrospective studies, such as the associations between *E. faecium* and hospital acquisition, previous use of antibiotics, especially penicillins and carbapenems, and a biliary tract source [4, 23, 24]. To our knowledge, the association between cerebrovascular disease or congestive heart failure with *E. faecalis* as opposed to *E. faecium* has not been previously reported, beyond the obvious association of *E. faecalis* with endocarditis and heart failure [6, 25].

Some studies have shown a higher CCI score for *E. faecium* BSI [5, 24]. In this study on the other hand and in a few others, the CCIs in patients with BSI were similar for both species [6, 23]. In addition, the reported average values of CCI vary widely between studies, probably reflecting considerable heterogeneity in the populations studied.

Overall, the significant differences in patient characteristics and sources of infection observed in this and previous studies indicate that BSI caused by *E. faecium* and *E. faecalis* are two distinct entities affecting different populations.

The observed in-hospital mortality rate of 20.8% for monomicrobial enterococcal BSI is consistent with previous studies [5, 7–10, 20, 22, 24, 26, 27]. In this study, we were unable to show a difference in in-hospital mortality between patients with *E. faecium* and *E. faecalis*. This is noteworthy, as multiple studies have shown higher fatality rates for *E. faecium* BSI. Two large population studies have shown mortality rates of 35% and 30% for *E. faecium* BSI vs 21% and 17% for *E. faecalis* BSI [5, 6]. Other, smaller studies have documented similar differences [9, 23, 27]. We hypothesise that this discrepancy could be due to multiple factors: firstly, unlike in some studies, average CCI scores were similar for patients with both species, a factor that is associated with mortality [28]; secondly, vancomycin-resistant *E. faecium,* which was previously shown as a negative prognostic factor*,*was rare in our population [7]. Of note, in Spain, the rate of vancomycin-resistant enterococci is low compared to other European countries [29]. However, some of the studies in which higher mortality was found for *E. faecium* cases also reported low vancomycin resistance rates [5, 6, 9, 27]. Importantly, active targeted antimicrobial therapy, a known protective factor for mortality, was higher in our cohort than in previous studies [1, 8, 22, 27], which might be related to the fact that most of the participating hospitals in our study had active bacteremia programs. Furthermore, congestive heart failure, a disease associated with a high mortality rate in the general population [30], was more prevalent among patients with *E. faecalis.* New studies, preferably prospective, are needed to determine whether mortality differs between the two species.

The analysis of mortality predictors confirmed some prognostic factors observed in earlier studies: age, CCI and clinical severity at BSI onset [5, 6, 9, 10, 20]. The association between urinary catheters and mortality has only been reported in a recent enterococcal BSI study [7]; interestingly, a strong association between urinary catheter use and in-hospital mortality has recently been shown for patients admitted to internal medicine wards, regardless of infection [31]. In our opinion, urinary catheter use would not have a direct causative role in mortality, but would be a surrogate variable related to the baseline condition of patients that is not captured by other variables. Further studies are warranted to assess this issue in patients with BSI.

Other studies have found that inappropriate targeted antimicrobial therapy is independently associated with mortality [8, 22, 27]. The association between active targeted therapy and mortality was also present in our bivariate analysis. Unfortunately, we could not include this into our multivariable model due to a substantial amount of missing data.

We found some differences in the prognostic factors for BSIs caused by *E. faecalis* and *E. faecium*. To our knowledge, the study by *Pinholt *et al. [6] is the only previously published study reporting species-specific independent predictors for mortality and showed conflicting results with our data. However, that study did not include some variables that were considered in our study, including specific comorbidities or severity of BSI. The fact that age or comorbidities were not associated with mortality in *E. faecium* BSI in our study may be due to lack of statistical power; however, the bivariate data suggest at least a lower impact of age and comorbidities compared to *E. faecalis*. In the *E. faecalis* group, urinary or biliary tract source was protective for mortality, as expected [32].

This study has several limitations that should be acknowledged. First, we did not include a control group without enterococcal BSI and therefore the predisposing factors studied are only useful to differentiate patients with one or the other species from those with enterococcal BSI. Second, to avoid the confounding effect of other bacteria in the mortality analyses, we had to exclude patients with polymicrobal bacteremia, which reduced the power of those analyses. Third, we used in-hospital mortality up to day 30 and may have missed some deaths that occurred after hospital discharge. Fourth, we did not perform molecular typing to characterize the molecular epidemiology of the isolates. Finally, this study only comprised Spanish hospitals. Consequently, the generalizibility to settings with different epidemiology and another level of access to healthcare is limited.

The strengths of the study are the large number of patients in the primary analysis and the prospective, multicenter design with cases from 26 hospitals in different regions of Spain, including community and university hospitals, which would make the study reasonably representative of the Spanish population. Finally, to our knowledge, this is one of the few studies that has analysed differences in predisposing and prognostic factors between patients with *E. faecium* and *E. faecalis* BSI.

In conclusion, BSI caused by *E. faecium* and *E. faecalis* are associated with different comorbidities and sources of infection. This study showed significant but similar in-hospital mortality rates for both pathogens, but while mortality in *E. faecium* BSI was associated only with severe BSI, *E. faecalis* BSI mortality was also associated with age, high comorbidity burden and source of infection. This study helps to understand the differences between these two pathogens in terms of patient population and prognostic factors and adds to the available data on enterococcal BSI.

## Supplementary Information

Supplementary Material 1.

## Data Availability

The data that support the findings of this study are available on request from the corresponding author.
