# Supplementary material for: Clinical characteristics, predisposing factors and outcomes for Enterococcus faecalis versus Enterococcus faecium bloodstream infections: a prospective multicentre cohort study
Source: Eur J Clin Microbiol Infect Dis. 2024 Aug 8;43(10):2011–22. doi: 10.1007/s10096-024-04917-5 (PMC11405461; doi:10.1007/s10096-024-04917-5)
Supplement: Supplementary file 1 — Supplementary Material 1. [file 10096_2024_4917_MOESM1_ESM.docx]

**Supplementary material**

**Table S1**: Multivariable analysis for predisposing factors and mortality in enterococcal bloodstream infection with data on multicollinearity.

**A.** Multivariable analysis for predictors of bloodstream infection due to E. faecium instead of E. faecalis.

|  | **B -coefficient** | **aOR (95% CI)** | **p-value** | **Collinearity statistic**  **VIF** |
| --- | --- | --- | --- | --- |
| Congestive heart failure^a^ | -0.671 | 0.51 (0.27 – 0.97) | **0.039** | 1.058 |
| Cerebrovascular disease^b^ | -0.783 | 0.45 (0.21 – 0.98) | **0.045** | 1.025 |
| Use of penicillins in previous month^c^ | 0.689 | 1.99 (1.20 – 3.32) | **0.008** | 1.060 |
| Use of carbapenems in previous month | 0.852 | 2.35 (1.12 – 4.93) | **0.025** | 1.082 |
| Hospital-acquired infection | 0.947 | 2.58 (1.61 – 4.12) | **<0.001** | 1.135 |
| Biliary source | 1.212 | 3.36 (1.84 – 6.13) | **<0.001** | 1.104 |
| Urinary source | -0.714 | 0.49 (0.26 – 0.92) | **0.028** | 1.084 |

aOR: adjusted OR, displayed as predisposing factors to *E. faecium*, in reference to *E. faecalis.*

*Legend*: ^a^Congestive heart failure: stage III or IV according to the New York Heart Association; ^d^Cerebrovascular disease: history of a cerebrovascular accident with minor/no residue or transient ischemic attacks. VIF: variance inflation factors

Area Under the Receiver Operating Curve: 0.74 (95% CI 0.70 - 0.79)

**B.** Monomicrobial enterococcal bloodstream infection

|  | **B-coefficient** | **aOR (95% CI)** | **p-value** | **Collinearity statistic**  **VIF** |
| --- | --- | --- | --- | --- |
| *E. faecium* (reference: *E. faecalis*) | 0.087 | 1.06 (0.55 - 2.07) | 0.861 | 1.013 |
| Age^1^ | 0.849 | 1.03 (1.00 – 1.06) | **0.031** | 1.016 |
| Charlson Comorbidity Index^2^ | 0.947 | 1.16 (1.03 – 1.31) | **0.013** | 1.091 |
| Urinary catheter | 0.845 | 2.69 (1.31 – 5.53) | **0.007** | 1.054 |
| SOFA score^2^ | 1.414 | 1.34 (1.17 – 1.53) | **<0.001** | 1.688 |
| Pitt score | 0.624 | 1.87 (0.93 – 1.26) | 0.304 | 1.630 |

**C.** Monomicrobial *E. faecalis* bloodstream infection

|  | **B-coefficient** | **aOR (95% CI)** | **p-value** | **Collinearity statistic**  **VIF** |
| --- | --- | --- | --- | --- |
| Age^1^ | 0.058 | 1.06 (1.02-1.10) | **0.004** | 1.018 |
| Charlson Comorbidity Index^2^ | 0.242 | 1.27 (1.08-1.51) | **0.005** | 1.084 |
| SOFA score^2^ | 0.383 | 1.47 (1.24-1.73) | **<0.001** | 1.085 |
| Urinary or biliary source | -1.239 | 0.29 (0.09-0.90) | **0.031** | 1.019 |

**C.** Monomicrobial *E. faecium* bloodstream infection

|  | **B-coefficient** | **aOR (95% CI)** | ***p-*value** | **Collinearity statistic**  **VIF** |
| --- | --- | --- | --- | --- |
| Age^1^ | -0.007 | 0.99 (0.95 - 1.03) | 0.731 | 1.029 |
| Charlson Comorbidity Index^2^ | -0.052 | 0.95 (0.79 – 1.14) | 0.586 | 1.153 |
| SOFA score^2^ | 0.294 | 1.34 (1.14 – 1.58) | **<0.001** | 1.021 |
| Urinary catheter | 1.082 | 2.95 (0.96 – 9.11) | 0.060 | 1.197 |
| Antibiotic use in previous month | 1.159 | 3.19 (0.96 – 10.60) | 0.059 | 1.226 |

*Abbreviations*: BSI: bloodstream infection; aOR: adjusted OR; SOFA: Sequential Organ Failure Assessment.

Area Under the Receiver Operating Curve: A: 0.81 (95% CI 0.75 - 0.87); B: 0.85 (0.78 - 0.93); C: 0.80 (0.70 - 0.90)

^1^Per year. ^2^Per index unit

**Table S2:**  General characteristics and outcomes of monomicrobial enterococcal BSI

|  | **All BSI**  **(n = 431)** | **E. faecalis BSI  (n = 267)** | **E. faecium BSI (n = 164)** | **p-value** |
| --- | --- | --- | --- | --- |
| **Demographic** |  |  |  |  |
| Age, median [IQR] | 71 [61 - 82] | 71 [62 - 82] | 69 [60 - 81] | 0.268 |
| Male sex, n (%) | 199 (65.2) | 123 (66.5) | 76 (63.3) | 0.572 |
| **Comorbidities, n (%)** |  |  |  |  |
| CCI, median (IQR) | 2 [1 – 5] | 3 [1-5] | 3 [0-5] | 0.645 |
| Congestive heart failure^a^ | 63 (20.5) | 48 (25.8) | 15 (12.4) | **0.004** |
| Diabetes Mellitus | 74 (24.1) | 47 (25.3) | 27 (22.3) | 0.554 |
| Chronic kidney disease^b^ | 49 (16.0) | 28 (15.1) | 21 (17.4) | 0.591 |
| Hepatic disease^c^ | 32 (10.4) | 16 (8.6) | 16 (13.2) | 0.195 |
| Solid tumor | 81 (26.4) | 23.1 (43) | 38 (31.4) | 0.107 |
| Cerebrovascular disease^d^ | 36 (11.7) | 26 (14.0) | 10 (8.3) | 0.128 |
| Hematologic malignancy | 20 (6.5) | 12 (6.5) | 8 (6.6) | 0.956 |
| Obstructive uropathy | 22 (7.2) | 18 (9.7) | 4 (3.3) | **0.034** |
| Obstructive biliary pathology | 16 (5.2) | 4 (2.2) | 12 (9.9) | **0.003** |
| Immunosuppressive therapy^e^ | 41 (13.4) | 25 (13.4) | 16 (13.2) | 0.956 |
| **Invasive procedures (in the previous month), n (%)** | |  |  |  |
| Surgery | 47 (15.3) | 20 (10.8) | 27 (22.3) | **0.006** |
| Bronchoscopy | 32 (10.4) | 13 (7.0) | 19 (15.7) | **0.015** |
| Urinary catheter | 66 (21.5) | 36 (19.4) | 30 (24.8) | 0.257 |
| **Use of antibiotics (in the previous month), n (%)** | |  |  |  |
| Any antibiotic | 126 (41.0) | 63 (33.9) | 63 (52.1) | **0.002** |
| Cephalosporins | 51 (16.6) | 27 (14.5) | 24 (19.8) | 0.221 |
| Penicillins | 66 (21.5) | 26 (14.0) | 40 (33.1) | **<0.001** |
| Carbapenems | 29 (9.4) | 6 (3.2) | 23 (19.0) | **<0.001** |
| Vancomycin, linezolid or daptomycin | 31 (10.1) | 13 (7.0) | 18 (14.9) | **0.025** |
| Quinolones | 39 (12.7) | 28 (15.1) | 11 (9.1) | 0.125 |
| **Type of acquisition, n (%)** |  |  |  |  |
| Hospital-acquired | 155 (52.2) | 66 (36.9) | 89 (75.4) | **<0.001** |
| Healthcare-associated | 78 (26.3) | 56 (31.3) | 22 (18.6) | 0.015 |
| Community-acquired | 64 (21.5) | 57 (31.8) | 7 (5.9) | **<0.001** |
| Onset in intensive care unit | 44 (14.7) | 18 (9.9) | 26 (21.8) | **0.004** |
| **Source of infection, n (%)** |  |  |  |  |
| Biliary tract | 36 (11.7) | 8 (4.3) | 28 (23.1) | **<0.001** |
| Abdominal (non-biliary) | 39 (12.7) | 18 (9.7) | 21 (17.4) | **0.048** |
| Catheter-related | 21 (6.8) | 13 (7.0) | 8 (6.6) | 0.898 |
| Endocarditis | 25 (8.1) | 23 (12.4) | 2 (1.7) | **<0.001** |
| Bone and joint | 0 (0.0) | 0 (0.0) | 0 (0.0) | NA |
| Skin and soft tissue | 5 (1.6) | 2 (1.1) | 3 (2.5) | 0.386* |
| Respiratory | 10 (3.3) | 9 (4.8) | 1 (0.8) | 0.095 |
| Central nervous system | 1 (0.3) | 1 (0.5) | 0 (0.0) | 1.000* |
| Urinary tract | 60 (19.5) | 49 (26.3) | 11 (9.1) | **<0.001** |
| Other | 3 (1.0) | 1 (0.5) | 2 (1.7) | 0.564* |
| Unknown | 107 (34.9) | 62 (33.3) | 45 (37.2) | 0.488 |
| **Microbiology, n (%)** |  |  |  |  |
| Ampicillin resistance^#^ | 101 (38.3) | 6 (3.7) | 95 (94.1) | **<0.001** |
| Vancomycin resistance^#^ | 8 (3.0) | 0 (0.0) | 8 (7.3) | **<0.001** |
| Active empirical antibiotic | 144 (46.9) | 102 (54.8) | 42 (34.7) | **<0.001** |
| Active targeted antibiotic ^#^ | 239 (93.7) | 140 (92.1) | 99 (96.1) | 0.195 |
| **Clinical presentation, n (%)** |  |  |  |  |
| Septic shock | 27 (8.8) | 12 (6.5) | 15 (12.4) | 0.072 |
| Pitt score, median [IQR] | 0 [0 – 3] | 0 [0– 3] | 0 [0 – 2] | 0.826 |
| SOFA score^#^, median [IQR] | 2 [0 – 4] | 2 [0– 4] | 2 [0 – 4] | 0.601 |
| **Outcome, n (%)** |  |  |  |  |
| In-hospital mortality | 64 (20.8) | 38 (20.4) | 26 (21.5) | 0.824 |
| BSI-related mortality | 35 (11.4) | 21 (11.3) | 14 (11.6) | 0.940 |
| Fever ≥ 72h | 54 (17.6) | 32 (17.2) | 22 (18.2) | 0.826 |
| Persistent BSI^f^ | 17 (5.5) | 11 (5.9) | 6 (5.0) | 0.721 |
| Recurrence of infection^g^ | 16 (5.2) | 6 (3.2) | 10 (8.3) | 0.052 |
| Readmission for recurrence | 9 (3) | 2 (1.1) | 6 (5) | **0.037** |

*Abbreviations* BSI: bloodstream infection; CCI: Charlson comorbidity index; NA: Not applicable. SOFA: Sequential Organ Failure Assessment.

*Legend*: ^a^Congestive heartfailure: stage III or IV according to the New York Heart Association; ^b^Chronic kidney disease: moderate to severe kidney disease, for more than 1 month; ^c^Hepatic disease: includes mild (chronic hepatitis) and severe (cirrhosis and portal hypertension with history of variceal bleeding) hepatic disease; ^d^Cerebrovascular disease: history of cerebrovascular accident with minor/no residual deficits or transient ischemic attacks; (continues next page)

Abbreviations (continued): ^e^Immunosuppressive therapy included antineoplastic chemotherapy and prednisone (or equivalent) at doses >10mg/day for more than 3 weeks; ^f^Persistent BSI: BSI with the same isolated organism on repeated blood culture > 72h from initial blood culture; ^g^Recurrence of infection: new BSI episode after achieving clinical cure during hospitalisation. ^h^Mortality: during hospitalisation, within a maximum of 30 days after the onset of BSI.

^#^Missing data: the following variables were available for a proportion of the patients: ampicillin resistance (n = 264/307); vancomycin resistance (n = 264/307) active targeted antibiotic (n = 255/307).

| A | BSI cases  (n = 307) | Mortality with factor | | Mortality without factor | | RR (95% CI) | *p* value | |
| --- | --- | --- | --- | --- | --- | --- | --- | --- |
| Demographic n (%) | | | | | | | | |
| Age ≥ 69 years | 170 (55.6) | 42/170 (24.7) | | 22/136 (16.2) | 1.52 (0.98 - 2.43) | | | **0.039^γ^** |
| Male sex | 199 (65.2) | 44/199 (22.1) | | 19/106 (17.9) | 1.23 (0.76 - 2.00) | | | 0.390 |
| Comorbidities, n (%) | | | | | | | | |
| CCI ≥ 3 | 151 (49.2) | 44/151 (29.1) | | 20/156 (12.8) | 2.27 (1.41 – 3.66) | | | **<0.001^γ^** |
| Congestive heart failure^a^ | 63 (20.5) | 20/63 (31.7) | | 44/244 (18.0) | 1.76 (1.12 - 2.76) | | | **0.017^γ^** |
| Diabetes mellitus | 74 (24.1) | 20/74 (27.0) | | 44/233 (18.9) | 1.43 (0.90 - 2.27) | | | 0.133**^γ^** |
| Chronic kidney disease^b^ | 49 (16.0) | 16/49 (32.7) | | 48/258 (18.6) | 1.75 (1.09 - 2.83) | | | **0.026^γ^** |
| Hepatic disease^c^ | 32 (10.4) | 12/32 (37.5) | | 52/276 (18.9) | 1.98 (1.19 - 3.30) | | | **0.014^γ^** |
| Peptic ulcer disease | 14 (4.6) | 7/14 (50.0) | | 57/293 (19.5) | 2.57 (1.45 - 4.57) | | | **0.013*^γ^** |
| Solid tumor | 81 (26.4) | 23/81 (28.4) | | 41/226 (18.1) | 1.56 (1.01 - 2.44) | | | 0.051^γ^ |
| Cerebrovascular disease^d^ | 36 (11.7) | 9/36 (25.0) | | 55/271 (20.3) | 1.23 (0.67 - 2.27) | | | 0.514 |
| Hematologic malignancies | 20 (6.5) | 5/20 (25.0) | | 59/287 (20.6) | 1.22 (0.55 - 2.69) | | | 0.636 |
| Hemiplegia | 16 (5.2) | 6/16 (37.5) | | 58/291 (19.9) | 1.88 (0.96 - 3.69) | | | 0.112^γ^ |
| Immunosuppressive therapy^e^ | 41 (45.9) | 12/41 (29.3) | | 52/266 (19.5) | 1.49 (0.88 - 2.56) | | | 0.154^γ^ |
| Invasive procedures (in the previous month), n (%) | | | | | | | | |
| Surgery | 47 (15.3) | 13/47 (27.7) | | 51/260 (19.6) | 1.41 (0.84 -2.38) | | | 0.212 |
| Urinary catheter | 66 (21.5) | 21/66 (31.8) | | 43/241 (17.8) | 1.78 (1.14 - 2.79) | | | **0.013**^γ^ |
| Antibiotic use in past month, n (%) | | | | | | | | |
| Any antibiotic | 126 (41.0) | | 33/126 (26.2) | 31/181 (17.1) | 1.53 (0.99 - 2.36) | | | 0.054^γ^ |
| Penicillins | 66 (21.5) | | 20/66 (30.3) | 44/241 (18.3) | 1.66 (1.05 - 2.61) | | | **0.033**^γ^ |
| Carbapenems | 29 (9.4) | | 9/29 (31.0) | 55/278 (19.8) | 1.57 (0.87 - 2.83) | | | 0.156^γ^ |
| Type of acquisition, n (%) | | | | | | | | |
| Hospital-acquired | 155 (52.2) | 37/155 (23.9) | | 26/142 (18.3) | 1.30 (0.83 - 2.04) | | | 0.242 |
| Healthcare-associated | 78 (26.3) | 13/78 (16.7) | | 50/219 (22.8) | 0.73 (0.42 - 1.27) | | | 0.253 |
| Community-acquired | 64 (21.5) | 13/64 (20.3) | | 50/233 (21.5) | 0.95 (0.55 - 1.63) | | | 0.842 |
| Onset in intensive care unit | 44 (36.4) | 16/44 (36.4) | | 48/256 (18.8) | 1.94 (1.22 - 3.10) | | | **0.008**^γ^ |
| Source of infection, n (%) | | | | | | | | |
| Biliary tract | 36 (11.7) | 8/36 (16.7) | | 58/271 (21.4) | 0.78 (0.36 - 1.68) | | | 0.511 |
| Abdominal (non-biliary) | 39 (12.7) | 10/39 (25.6) | | 54/268 (20.1) | 1.72 (0.71 - 2.28) | | | 0.430 |
| Catheter-related | 21 (6.8) | 6/21 (28.6) | | 58/286 (20.3) | 1.41 (0.69 - 2.88) | | | 0.403* |
| Skin and soft tissue | 5 (1.6) | 2/5 (40.0) | | 62/302 (20.5) | 1.95 (0.65 - 5.85) | | | 0.280* |
| Respiratory | 10 (3.3) | 3/10 (30.0) | | 61/297 (20.5) | 1.46 (0.55 - 3.86) | | | 0.440* |
| Central nervous system | 1 (0.3) | 0/1 (0.0) | | 64/306 (20.9) | NA | | | 1.000* |
| Endocarditis | 25 (8.1) | 5/25 (20.0) | | 59/282 (20.9) | 0.96 (0.42 - 2.16) | | | 0.913 |
| Urinary tract | 60 (19.5) | 11/60 (18.3) | | 53/247 (21.5) | 0.85 (0.48 - 1.53) | | | 0.593 |
| Other | 3 (1.0) | 0/0 (0.0) | | 64/304 (21.1) | NA | | | 1.000* |
| Unknown | 107 (34.9) | 21/107 (19.6) | | 43/200 (21.5) | 0.91 (0.57 - 1.46) | | | 0.700 |
| Etiology | | | | | | | |  |
| *E. faecalis* | 186 (60.6) | 38/186 (20.4) | | 26/121 (21.5) | 0.95 (0.61 - 1.48) | | | 0.824^γ^ |
| *E. faecium* | 121 (39.4) | 26/121 (21.5) | | 38/186 (20.4) | 1.05 (0.68 - 1.64) | | | 0.824^γ^ |
| Ampicillin resistance^#^ | 101 (38.2) | 22/101 (21.8) | | 31/163 (19.0) | 1.45 (0.70 - 1.86) | | | 0.586 |
| Vancomycin resistance^#^ | 8 (3.0) | 1/8 (12.5) | | 53/255 (20.8) | 0.60 (0.09 - 3.82) | | | 1.000* |
| Active empirical treatment | 144 (46.9) | 34/144 (23.6) | | 30/163 (18.4) | 1.28 (0.83 - 1.98) | | | 0.262 |
| Active targeted treatment^#^ | 239 (93.7) | 40 (16.7) | | 6/16 (37.5) | 0.45 (0.22 – 0.89) | | | **0.047*** |
| Clinical presentation, n (%) | | | | | | | |  |
| Septic shock | 27 (8.7) | 14/27 (51.9) | | 50/280 (17.9) | 2.91 (1.87 - 4.52) | | | **<0.001**^γ^ |
| Pitt score ≥ 3 | 75 (24.4) | 29/75 (38.7) | | 35/232 (15.1) | 2.56 (1.69- 3.89) | | | **< 0.001**^γ^ |
| SOFA ≥ 3 | 125 (41.5) | 43/125 (34.4) | | 16/176 (9.1) | 3.79 (2.24 - 6.41) | | | **<0.001**^γ^ |
|  |  |  | |  |  | | |  |

**Table S3:** Bivariate analysis in-hospital mortality for monomicrobial enterococcal bloodstream infection. A) general characteristics; B) data disease progression and outcomes.

Abbreviations: BSI: blood stream infection; CCI: Charlson Comorbidity Index; NA: Not applicable; SOFA: Sequential Organ Failure Assessment.

*p*-value: * calculated by Fisher’s exact test, all other p-values are calculated by chi-square (Continues next page).

| B | BSI cases  (n = 307) | Mortality with factor | Mortality without factor | RR (95% CI) | *p* value |
| --- | --- | --- | --- | --- | --- |

| Disease progression, n (%) | | | | | |
| --- | --- | --- | --- | --- | --- |
| Fever ≥ 72 h | 54 (17.6) | 17/54 (31.5) | 47/253 (18.6) | 1.69 (1.06 - 2.71) | **0.034** |
| Infected endovascular material | 16 (5.2) | 4/16 (25.0) | 60/291 (20.6) | 1.21 (0.50 -2.92) | 0.674 |
| Persistent BSI^f^ | 17 (5.5) | 5/17 (29.4) | 59/290 (20.3) | 1.45 (0.67 - 3.13) | 0.371 |
| Recurrence of infection^g^ | 16 (5.2) | 2/16 (12.5) | 62/291 (21.3) | 0.59 (0.16 - 2.18) | 0.399 |
| Readmission for recurrence | 9 (3) | 0 (0) | 64/307 (20.8) | - | 0.099 |
| Outcome, n (%) |  |  |  |  |  |
| In-hospital mortality | 64 (20.8) | NA | NA | NA | NA |
| BSI-related mortality | 35 (11.4) | NA | NA | NA | NA |

Definitions (continued): ^a^Congestive heart failure: stage III or IV according to the New York Heart Association; ^b^Chronic kidney disease: moderate to severe kidney disease, for more than 1 month; ^c^Hepatic disease: comprises mild (chronic hepatitis) and severe (cirrhosis and portal hypertension with history of variceal bleeding) hepatic disease; ^d^Cerebrovascular disease: history of a cerebrovascular accident with minor/no residual deficit or transient ischemic attacks (continues next page) ^e^Immunosuppressive therapy: cancer chemotherapy or radiotherapy, typical immunosuppressants, and steroids if dosing used was >10 mg/day of prednisone or equivalent for more than 3 weeks; ^f^Persistent BSI: BSI with the same organism isolated in repeated blood culture > 72h from initial blood culture; ^g^Recurrence of infection: new BSI episode after achieved clinical cure during hospitalization. ^γ^ Variables used with stepwise-backward multivariabe analysis. In addition to these variables, recurrent urinary tract infection (p 0.078) and colonoscopy in previous month (p 0.193*) were also used in the initial model of the multivariable analysis

^#^Missing data: the following variables were available for a proportion of the patients: ampicillin resistance (n = 264/307); vancomycin resistance (n = 264/307); active targeted treatment (n = 255/307).
